# Electrostatic Induction Nanogenerator Boosted by One‐Dimensional Metastructure: Application to Energy and Information Transmitting Smart Tag System

**DOI:** 10.1002/advs.202205141

**Published:** 2023-01-22

**Authors:** Geon‐Ju Choi, Sang‐Hyun Sohn, Il‐Kyu Park

**Affiliations:** ^1^ Department of Materials Science and Engineering Seoul National University of Science and Technology Seoul 01811 Republic of Korea

**Keywords:** electrostatic induction, metastructure, nanogenerator, polyvinylidene fluoride, ultrasound wave

## Abstract

The recent application of the internet of things demands the ubiquitous utilization of data and electrical power. Even with the development of a wide variety of energy‐harvesting technologies, few studies have reported a device transporting electrical energy and data simultaneously. This paper reports an electrostatic induction nanogenerator (ESING) consisting of a one‐dimensional metastructure that can modulate the output voltage based on the resonance of ultrasound waves to transmit energy and data simultaneously. The ESING device is fabricated using electronegative poly(vinylidene fluoride) (PVDF) membrane using a phase inversion process. The output voltage from the ESING device exhibits periodic resonant peaks as the gap between the PVDF membrane and the Al electrode changes, showing an up to 35‐fold difference between the maximum and minimum output voltages depending on the resonance state. The energy and electrical signal can be transmitted simultaneously in free space because the ESING converts energy from high‐frequency ultrasound waves. This paper provides proof of concept for a data and energy‐transferable smart tag device based on ESING devices exhibiting resonant and non‐resonant states. A device consisting of four ESINGs for a 4‐bit signal is implemented to demonstrate 16 signals.

## Graphical abstract

An electrostatic induction nanogenerator is demonstrated based on a one‐dimensional metastructure, which boosts the output voltage up to 35 times. The electrical energy and signal can be transferred simultaneously in free space. Proof of concept for a data and energy‐transferable smart tag device is demonstrated based on the nanogenerator exhibiting resonant and non‐resonant states for a 4‐bit signal.

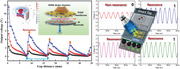

## 1 Introduction

Recently, ubiquitous energy and information utilization have attracted considerable attention for various applications on internet of things (IoT) technology. The sustainable energy harvesting of wasted energy from the environment also has become increasingly important because of the tremendous number of devices required to realize wireless sensor networks and the depletion of energy resources. One of the solutions to these problems is mechanical energy harvesting technology that converts waste mechanical energy into usable electrical energy.^[^ ^1^, ^2^, ^3^, ^4^ ^]^ The mechanical energy harvesting technology converts waste mechanical energy sources, such as physical movement, frictional motion, vibration, or sound waves, into electrical energy based on piezoelectric, triboelectric, and electrostatic induction mechanisms.^[^ ^5^, ^6^, ^7^, ^8^, ^9^, ^10^, ^11^, ^12^, ^13^, ^14^ ^]^ Although various piezoelectric and triboelectric energy harvesting technologies have been used in many practical applications, there is a need to improve the reliability and output performance.^[^ ^12^, ^13^, ^14^ ^]^ Physical contact between the active materials degrades the output performance during the operation and the long‐term stability.^[^ ^6^, ^7^ ^]^ To avoid the performance degradation of triboelectric or piezoelectric nanogenerators during long‐term operation, devices operating without physical friction or contact have been investigated by using an air gap or liquid.^[^ ^15^, ^16^, ^17^ ^]^ Electrostatic induction can also circumvent these problems because it induces the directional movement of electrons when ferroelectric or charged objects approach each other.^[^ ^10^ ^]^ Unlike piezoelectric or triboelectric mechanisms, the electrostatic induction mechanism does not require physical contact to produce electrical energy. In addition, it can convert energy even from tiny amounts of mechanical energy, such as sound waves. The sound wave energy source is clean energy found around the human body regardless of time and position. Therefore, energy‐harvesting techniques utilizing such ambient sound energy have been actively investigated.^[^ ^18^, ^19^, ^20^, ^21^, ^22^, ^23^, ^24^ ^]^

**Figure 1:**
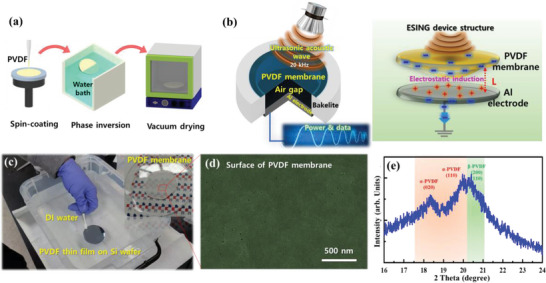
Preparation of the PVDF membrane and its structural property. a) Schematic illustrations of the fabrication process of the PVDF membrane; Spin‐coating, phase inversion, and vacuum‐drying. b) Schematic diagram of the ESING device structure and its operating principle. c) Photograph of the delamination of the PVDF thin film membrane from the Si wafer by the phase‐inversion process. d) FESEM image of PVDF membrane surface. e) XRD pattern of the PVDF membrane.

Ultrasound with a frequency above 20 kHz is relatively harmless to the human body. In addition, the waveform of sound energy can be resonated to amplify the sound pressure using metastructures, such as a Helmholtz resonator or a quarter‐wavelength resonator using various piezoelectric materials.^[^ ^25^, ^26^, ^27^, ^28^, ^29^, ^30^ ^]^ Therefore, energy harvesting performances can be enhanced by combining an electrostatic induction mechanism and resonance of ultrasound waves. Nevertheless, there have been few investigations on electrostatic induction‐based energy and information transfer technology. This paper reports an ultrasound wave‐stimulating electrostatic induction nanogenerator (ESING) using a one‐dimensional metastructure fabricated by controlling the gap between an electronegative polyvinylidene fluoride (PVDF) membrane and an aluminum (Al) electrode. The thickness‐controlled PVDF membrane with a high fraction of polar *β*‐phase was fabricated using a phase inversion process as shown in **Figure** **1**a.^[^ ^31^ ^]^ Based on the ultrasonic wave resonating between the PVDF membrane and the Al electrode, the output voltage of the ESING device could be boosted considerably and modulated to demonstrate a proof‐of‐concept smart tag system for data and energy transfer(Figure 1a). The electrical power transferred from the ESING device could be stored in the capacitor to operate the smart tag system or turn on light‐emitting devices. In this way, the stored electrical power in the smart tag through the ESING can be used as data as itself or to power up some other devices.

## 2 Results and Discussion

The PVDF membrane fabricated by the phase inversion process had a relatively flat and uniform morphology as shown in Figure 1c,d. The field‐emission scanning electron microscope (FESEM) image of the delaminated PVDF surface revealed a very smooth surface without pores and lumps (Figure 1d). The X‐ray diffraction (XRD) pattern of the delaminated membrane revealed a typical crystalline PVDF structure, showing two peaks corresponding to the *α*‐ and *β*‐phases (Figure 1e). The performance of the ESING improved as the *β*‐phase ratio increased because it possesses higher polarity. The intensity of the *β*‐phase peak (20.5° 2*θ*) is larger than that of the *α*‐phase (18.5° 2*θ*). This indicates that the PVDF membrane exhibits a high *β*‐phase ratio.^[^ ^31^ ^]^

The 20 kHz ultrasound wave was incident to the device to convert the ultrasound energy into electrical energy and signals using the ESING device, as shown schematically in Figure 1a. As the ultrasound wave was transferred to the surface of the ESING device, the PVDF membrane vibrated by being deflected back and forth. Electrons were repelled from the Al electrode as the electronegative PVDF membrane approached the Al surface. This way, the ultrasound wave can be converted to electrical energy and signals from the ultrasound source to the ESING.

**Figure 2:**
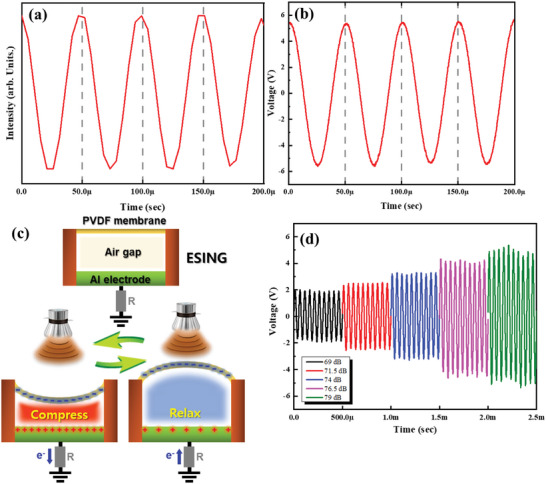
Operating principle of the ESING device. a) Frequency of the 20 kHz ultrasound source measured using a microphone. b) The output voltage of the ESING device when an ultrasound wave with 79 dB intensity is applied. c) Schematic diagram showing the operating principle of the ESING device: vibration of the PVDF membrane and the change in internal pressure when the ultrasound wave is incident. d) The output voltage of ESING as a function of the ultrasound intensity from 69 to 79 dB.

The output signal of the ultrasound source was measured using a commercial microphone to confirm the transferred waveform. **Figure** **2**a shows a waveform of the ultrasound source measured using a microphone with a sampling rate of 192 000. The measured period was 50 µs, confirming that the 20 kHz ultrasound wave with a sinusoidal waveform was well generated. As shown in Figure 2b, the output voltage was measured by setting the intensity of the ultrasound source and the gap between the PVDF membrane and electrode to 79 dB and 0.5 mm, respectively. The output voltage was ≈11.6 V_pp_ and the period was 50 µs. The ESING device follows the high‐frequency input signal of the ultrasound source. Figure 2c shows the working principle of ESING. As the ultrasound wave is incident to the ESING device, the PVDF membrane vibrates, and the air pressure between the membrane and the Al electrode changes. As the PVDF membrane approaches the Al electrode, the free electrons are pushed out from the Al electrode. The Al electrode is then positively charged. As the PVDF membrane is relaxed and recedes from the Al electrode, the free electrons return to the Al electrode to maintain charge neutrality. Even though the ESING device was not treated for any operation, the static charge on the PVDF surface was maintained during the operation of the ESING under ultrasonic exposure. This would be due to the interaction between the PVDF surface and the colliding air molecules to form a permanently polarized electret.^[^ ^32^ ^]^

Here, the principle that the wavelength of the ultrasound wave matches the wavelength of output voltage harvested by ESING can be explained as follows. The time‐dependent distance between the PVDF membrane and the Al electrode due to the ultrasound wave‐induced vibration is expressed using the following equation:

(1) $$s\left(t\right)\;=s_{0}\;+a\sin\omega t$$

where *s*_0_ is the original gap spacing; *a* is the amplitude of the vibration; *ω* is the oscillation frequency. At this time, the charge density induced at the Al electrode can be expressed as follows:

(2) $$\sigma_{\mathrm{Al}}=\;\frac{d\sigma_{\mathrm{PVDF}}}{\varepsilon_{r}\left(s_{0}+a\sin\omega t\right)+d}$$

where *σ*_PVDF_ is the charge density at the PVDF surface; *d* is the thickness of the membrane; *ε*_r_ is the dielectric constant. A current is generated through the external circuit because *σ*_Al_ varies with time. The current *I* is expressed as

(3) $$I\;=\;A\;\frac{d\sigma_{\mathrm{Al}}}{dt}=\frac{Ada\varepsilon_{r}\omega \sigma_{\mathrm{PVDF}}}{{\left[\varepsilon_{r}\left(s_{0}+a\sin\omega t\right)+d\right]}^{2}}\;\cos\omega t$$

where *A* is the area of the vibrating PVDF surface. Because *a* is a relatively a much small value than *s*_0_, the output voltage (*V*_R_) of the ESING device when a load resistor *R* is connected can be expressed as

(4) $$V_{R}=\;IR\;=\frac{Ada\varepsilon_{r}\omega \sigma_{\mathrm{PVDF}}R}{{\left(\varepsilon_{r}s_{0}+d\right)}^{2}}\;\cos\omega t$$

**Figure 3:**
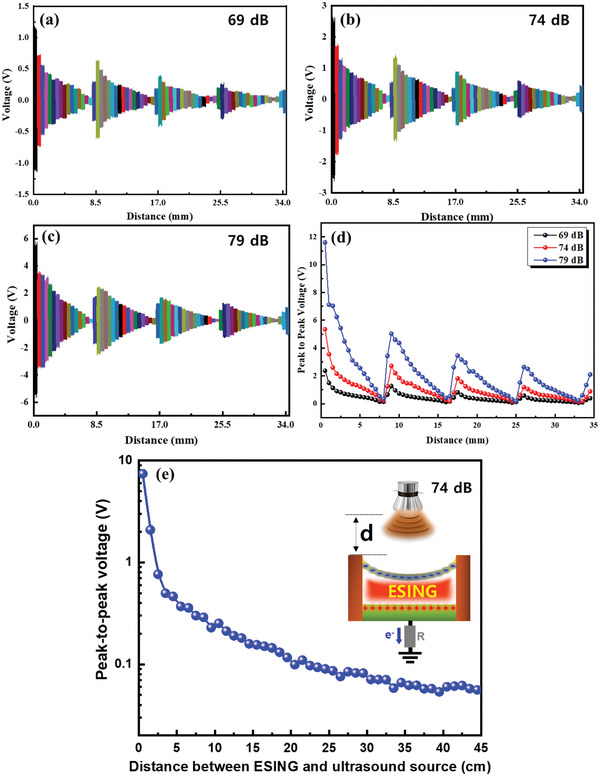
Parametric study of ESING as a function of various factors. The output voltage of the ESING device with the variation of the gap between the PVDF membrane and the Al electrode from 0.5 to 36 mm for various ultrasound intensities: a) 69 dB, b) 74 dB, and c) 79 dB. The output voltage signals with variations of the gap were shown in different colors. d) Peak‐to‐peak output voltage varies with the gap between the PVDF membrane and the Al electrode. e) Output voltage of ESING according to the distance between the ultrasonic source and the device ranging from 1 to 45 cm. The ultrasonic intensity was 74 dB.

Applying this equation to the present ESING device, *A, d, a, ε*_r_, *s*_0_, and *R* are 0.01^2^ *π* m^2^, 10^−5^ m, 10^−4^ m, 12, 0.009 m, and 100 MΩ, respectively. A surface DC voltmeter measured *σ*_PVDF_ to be 7.91 × 10^−5^ C m^−2^. The value obtained by substituting this revealed a numerical value similar to the experimental data. (Figure S1, Supporting Information). Therefore, the time‐dependent output voltage can follow the incident ultrasound wave as shown in Figure 2a,b. The change in performance of the ESING device according to the intensity of the ultrasound source was confirmed by changing the intensity of the ultrasound source from 69 to 79 dB and measuring the output voltage (Figure 2d). The amplitude of the variation(*a*) increased as the ultrasound source intensity increased, which resulted in a linear increase in the output voltage without showing a difference in the waveform. This suggests that the ESING device can also be applied to information transfer via an amplitude modulation (AM) mode. If the gap between the PVDF membrane and the Al electrode matches an integer multiple of the half‐wavelength of the ultrasound wave, the PVDF membrane experiences greater displacement due to the larger pressure by the constructive interference of the incoming and outgoing ultrasound waves. This resulted in an enhanced output voltage of the ESING device by constituting a one‐dimensional metastructure. The resonance behavior of the metastructure was examined by adjusting the gap between the PVDF membrane and the Al electrode and measuring the output voltage. **Figure** **3**a–c shows the output voltage as a function of the distance between the PVDF membrane and the Al electrode with various ultrasound intensities (69, 74, and 79 dB). For all ultrasound intensities, the output voltage of the ESING device exhibited periodic variations with increasing gap distance, indicating resonant and non‐resonant behaviors. As the gap between the PVDF membrane and the Al electrode of the ESING device increased, the output voltage increased and showed a maximum value at certain distances. The peak output voltage decreased gradually with increasing gap because of the reduced deflection of the PVDF membrane, as shown in Figure 3d. The specific distance was formed at ≈8.5 mm intervals, which matched well with a half‐wavelength of the ultrasound wave with a frequency of 20 kHz. The speed of the ultrasound wave at 25 °C is ≈346.5 m s^−1^. Hence, the wavelength of the incident ultrasound of 20 kHz was calculated as follows:

(5) $$346.5\;\left({m\;s}^{-1}\right)\times \frac{1}{20000}\;\;\left(s\right)=\;0.017325\;m$$

Resonance occurs in the inner space at half‐wavelength intervals of ≈8.662 mm because the ultrasound wavelength is 17.325 mm. At every 8.622 mm, the deformation of the PVDF membrane would be maximized because of constructive interference, resulting in the maximum output voltage. The maximum output voltage was ≈11.6 V under constructive interference conditions, dropping to 0.33 V under destructive interference conditions. The maximum and minimum output voltages showed an ≈35‐fold difference by adjusting the gap between the PVDF membrane and Al electrodes. In addition, the output voltages were tens of times larger than the minimum values at all the resonant positions. This suggests that two or more voltage signals with different intensities can be formed by simply adjusting the gap of the ESING device. In addition, the output voltage from the ESING device decreased with increasing distance from the ultrasound source because of the distance‐dependent sound intensity attenuation, which follows an inverse square law (Figure 3e).

**Figure 4:**
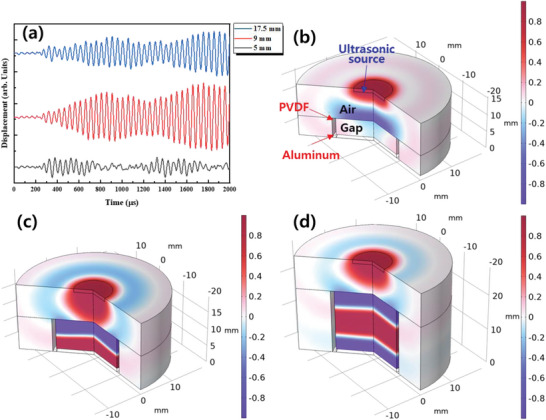
Data analyzed with COMSOL Multiphysics. a) Displacement of PVDF membrane with time when the gap between the PVDF membrane and Al electrode is 5, 9, and 17.5 mm. Pressure distribution at the time of 2000 µs for the different gaps: b) 5 mm, c) 9 mm, and d) 17.5 mm.

The displacement of the PVDF membrane was analyzed theoretically to assess the resonant behavior. **Figure** **4**a shows the time‐dependent displacement of the PVDF membrane when a 20 kHz ultrasound wave was applied for various gaps between the PVDF membrane and the Al electrode. The gap values were set to 5, 9, and 17.5 mm. The wavelength of the sound wave with a 20 kHz frequency was very close to 17.5 mm, and the half‐wavelength was ≈9 mm. The displacement lost periodicity at around 700 and 1800 µs when the gap was 5 mm. In this case, the periodicity is broken due to destructive interference because the distance between the membrane and the Al electrode does not correspond to an integer multiple of the half‐wavelength of 20 kHz (Videos S1–S4, Supporting Information). When the gaps were 9 and 17.5 mm, however, the displacement showed periodicity while increasing the intensity gradually. Figure 4b–d shows the simulated pressure distributions at 2000 µs at gaps of 5, 9, and 17.5 mm, respectively. In the case of a 5 mm gap, the pressure in the space between the PVDF membrane and the Al electrode was much lower than that near the ultrasound source (Figure 4b). As the gap was increased to 9 and 17.5 mm, high‐pressure regions were formed due to the constructive interference of the incident and retracing pressure waves between the PVDF membrane and the Al electrode (Figure 4c,d).

**Figure 5:**
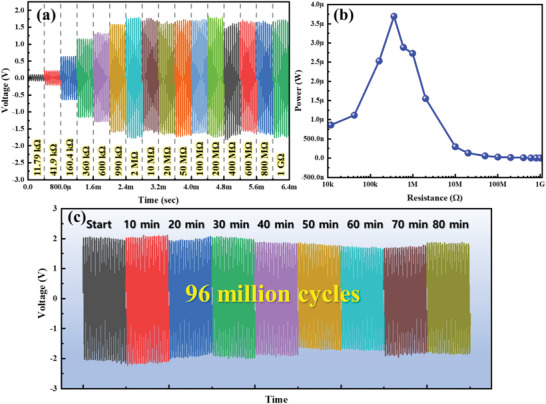
Parametric study of ESING as a function of various factors. Load resistance‐dependent a) output voltage and b) output power of ESING. The ultrasound intensity was 74 dB, and the gap between the PVDF membrane and the Al electrode was 9 mm. c) Durability data measuring the output voltage of ESING during operation for 80 min measured every 10 min.

The detailed performance of the ESING device was examined by measuring the load resistance‐dependent output voltage at different resistances from 11.79 kΩ to 1 GΩ, as shown in **Figure** **5**a. The intensity of the ultrasound source and the gap between the PVDF and Al electrodes were set to 74 dB and 9 mm, respectively. The output voltage increased at the low‐resistance range under 2 MΩ, decreasing slightly with further increases in resistance. This behavior was similar to triboelectric or piezoelectric nanogenerators.^[^ ^5^, ^6^, ^8^, ^9^ ^]^ The output power was estimated based on $P\;=\;\frac{V^{2}}{R}$ from the load resistance‐dependent output voltage results, as shown in Figure 5b. The maximum output power was 3.7 µW at 360 kΩ. According to the built‐capacitive model, the ESING device can be considered an open‐circuit voltage source in series with a time‐varying capacitor, which serves as a resistor to the flow of electric charges and contributes to the internal resistance. The maximum electrical energy would be obtained when the external load is equal to the internal resistance of the ESING device. Therefore, the corresponding resistance of the ESING device is ≈360 kΩ.

The reliability of mechanical energy‐based nanogenerators is crucial because of the physical damage to the active materials during operation. This study investigated the reliability of the proposed ESING device by operating it for more than 80 min. Figure 5c shows the output voltage of the ESING according to the operation time with the continuous incidence of the ultrasound wave. The output voltage was maintained at the initial values and shapes without showing significant degradation for up to 80 min. This indicates that the ESING device operated more than 96 000 000 cycles considering the ultrasound wave with a 20 kHz frequency. This confirms the long‐term durability of the ESING device.

**Figure 6:**
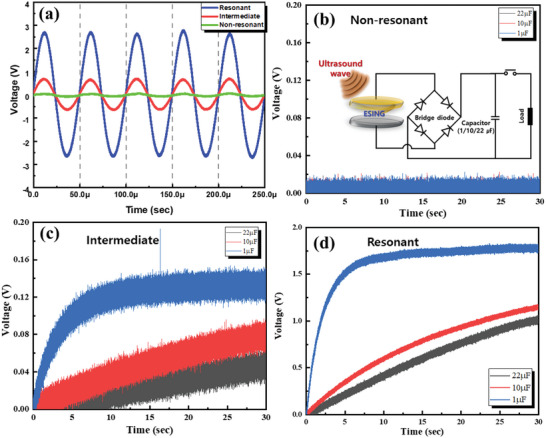
Parametric study of ESING as a function of various factors. a) The difference in the output voltage between the ESING devices with resonant (constructive interference), non‐resonant (destructive interference), and intermediate conditions by adjusting the gap between the PVDF membrane and the Al electrode. Charging performance by connecting capacitors with capacitances of 1, 10, and 22 µF to ESING devices operating under different conditions: b) non‐resonant, b) intermediate, and c) resonant conditions. The inset shows the equivalent circuit diagram.

The charging performance of the ESING devices was investigated depending on the resonant conditions. The gap of the ESING device was set to three conditions: 9 mm (a resonant condition where constructive interference occurs), 5 mm (intermediate condition), and 8 mm (a non‐resonant condition where destructive interference occurs). **Figure** **6**a shows the time‐dependent output voltages of the ESING devices operating under three conditions. As expected theoretically, the ESING device with a resonant gap showed an output voltage of 2.7 V, which is ≈45 times larger than the non‐resonant gap (Figure 6a). These three ESING devices were connected to 1, 10, and 22 µF capacitors. They were rectified through a bridge diode because the ESING device generates an alternating current (AC), as shown in the inset of Figure 6b. Figure 6b–d shows the charging curves of the capacitors by the ESING under three conditions. The ESING with the non‐resonant gap (8 mm) showed a negligible voltage with virtually no charge (Figure 6b). The voltage did not increase, even when the charging time was increased to 30 s, because of the negligible output voltage. The capacitor voltage was increased slightly by adjusting the gap (5 mm) to avoid the non‐resonant condition, but the saturation voltage value was still very small. On the other hand, the ESING with a resonant gap charged the capacitors entirely within 10 s with a sinusoidal output voltage of 20 kHz, as shown in Figure 6d. Although the ESING device generated a relatively lower output voltage than triboelectric nanogenerators, it allowed rapid charging of the capacitors quickly because it harvests energy from high‐frequency ultrasound energy.

**Figure 7:**
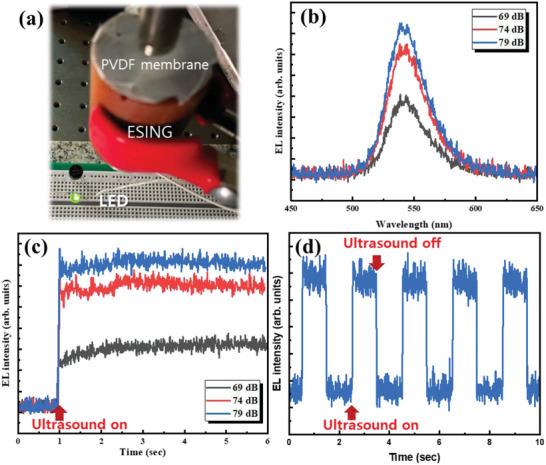
Applications of the ESING. a) ESING device operated by ultrasound continuously powers a commercial green LED lamp. b) EL spectra of a green LED lit by the ESING device operated at various ultrasound intensities. c) Time‐dependent EL intensity of the green LED powered by the ESING device with various ultrasound intensities. d) Time‐dependent EL intensity of the green LED for the ultrasound pulses with 1‐s intervals.

The ESING also continuously harvested the energy through free space and powered passive devices, such as light‐emitting diodes (LED). Therefore, the ESING under the incident ultrasound wave could continuously drive the commercial green LED without connecting the charge storage devices (**Figure** **7**a and Video S5, Supporting Information). Figure 7b shows the electroluminescence (EL) spectra of the green LED powered by the ESING with the variation of ultrasound intensity from 69 to 79 dB. The EL intensity of the LED increased continuously with increasing incident ultrasound intensity. When powering the LED with the ESING, the variation of the EL intensity was monitored with time (Figure 7c). The LED operated continuously without flickering because the ESING generated electrical energy continuously from the incident ultrasound wave. The intensity of the LED increased with ultrasound intensity due to the increased output voltage of the ESING. Figure 7d presents a graph showing the EL intensity of the LED lit by the ESING as the ultrasound is incident with pulses at 1‐s intervals. The transient behavior of the LED matched with the incident ultrasound wave source through the ESING device. This suggests that the ESING device can be applied to a wireless power and data transmission system by continuously supplying electrical energy.

**Figure 8:**
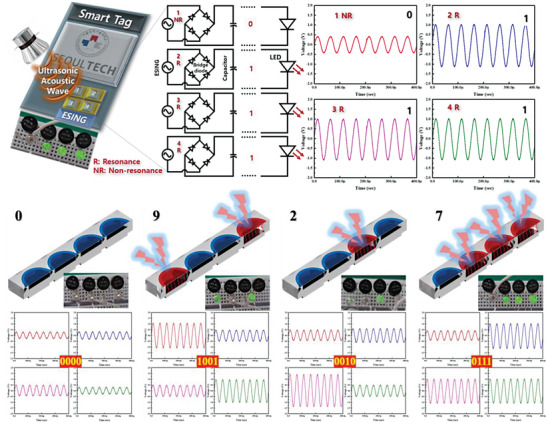
Application demonstration of smart tag system‐based ESING in various resonant states. Schematic illustration and equivalent circuit diagram of the smart tag system consisting of four ESING devices transmitting electrical power and information simultaneously. The tag system consisted of four ESING devices to demonstrate 4‐bit signals. The green LED array can be turned on by connecting the smart tag already programmed by the ESINGs. Four numbers of 0, 9, 2, and 7 are expressed by the 4‐bit signals of 0000, 1010, 0010, and 0111, respectively.

The ESING devices can transmit electrical power and information simultaneously through free space, extending the benefits to various applications and realizing wireless sensor networks. The higher the ultrasound frequency, the faster the energy and information that the ESING can transfer. To this end, a proof‐of‐concept was demonstrated for the data and electrical power storage and transfer tag consisting of four ESING devices, as shown in **Figure** **8**. As described above, the output voltage of the ESING device can be modulated by controlling the resonant distance, and the modulated voltage can be applied to a multi‐bit data storage tag. The digits of “one” and “zero” could be distinguished by the resonant and non‐resonant conditions of the ESING device, respectively. The case in which a non‐resonant condition generated a low output volage was set to “zero.” In contrast, a double output voltage generated by the resonant condition was set to “one.” Four ESING devices act as a cell to store data and provide a power source. Different output voltages were generated from a single ultrasound source because the gap between the PVDF membrane and the Al electrode of each ESING is individually adjustable. By modulating the resonance (R) and non‐resonance (NR) states of the four ESINGs, the 4‐bit signal tag system could express 16 numbers from 0 to 15. For example, four numbers of 0, 9, 2, and 7 were demonstrated as signals of 0000, 1010, 0010, and 0111, respectively (Figure 8). Hence, electrical power and data could be stored and transferred by connecting four capacitors to the ESING devices constituting a cell. Therefore, a one‐dimensional metastructure based on a vibrating PVDF membrane can be applied to an ESING device, which can harvest ultrasound waves and transmit information simultaneously through free space.

## 3 Conclusions

An energy‐harvesting and information‐transporting ESING device was demonstrated based on an electrostatic induction mechanism stimulated by an ultrasound wave. The output voltages of the ESING devices were boosted using a one‐dimensional metastructure, which modulated the resonant and non‐resonant states by controlling the gap between the electronegative PVDF membrane and the counter metal electrode. The output voltage of the ESING device showed periodic resonant behavior with variations of the gap by forming a one‐dimensional metastructure. The output voltage could be boosted up to 35‐fold compared to the non‐resonant one by controlling the resonant gap spacing. Because the ESING device harvested energy from high‐frequency ultrasound, it could charge the capacitors quickly and continuously, sufficient to power a commercial green LED. Finally, a data and energy‐transferable smart tag system was demonstrated using four ESING devices with resonant and non‐resonant states. The smart tag showed 16 numbers using a 4‐bit signal.

## 4 Experimental Section

### Fabrication of PVDF Membrane

The PVDF membrane was fabricated using a phase inversion process, as illustrated in Figure 1a. The PVDF source solution was prepared by dissolving a 15 wt% PVDF pellet (MW = 275 000, Sigma‐Aldrich) in *N*,*N*‐dimethylformamide (DMF, Mallinckrodt chemicals). The solution was stirred at 70 °C for 4 h. After preparing the solutions, the PVDF was spin‐coated on a 2 in. silicon wafer at 4000 rpm for 30 s. Under this condition, the thickness of the PVDF thin film was ≈10 µm. The PVDF‐coated wafer was immersed immediately into a DI water bath at 20 °C to induce phase inversion for delamination of the PVDF membrane from the supporting substrate.^[^ ^33^, ^34^, ^35^, ^36^ ^]^ The PVDF membrane was delaminated spontaneously from the silicon wafer within a few seconds using a phase inversion process, as shown in Figure 1b. The separated PVDF membrane was dried in a vacuum oven at 25 °C for 24 h.

### Fabrication of the ESING Devices

As shown in Figure 1a, the PVDF membrane was attached to a Bakelite cylinder with a hole diameter of 20 mm. The PVDF membrane was used without additional poling or charging processes. The aluminum (Al) electrode was inserted into the opposite cylindrical hole. The air gap between the PVDF membrane and the Al electrode was adjusted precisely to determine the resonant distance.

### Theoretical Design and Analysis

COMSOL Multiphysics 5.6 was used to analyze the resonance phenomenon according to the frequency of ultrasound waves within the gap between the PVDF membrane and the Al electrode using the finite element method. For analysis with an axisymmetric shape, the device structure was modeled using Revolution 2D. The PVDF membrane was set to a diameter of 20 mm and a thickness of 10 µm. The ultrasound source was positioned 8.5 mm away from the PVDF membrane. Pressure Acoustics and Solid Mechanics were used for the physics. The Acoustic‐Structural boundary was used for Multiphysics (COMSOL). The boundary conditions of Pressure Acoustics were set as follows. The pressure (*P*) of the ultrasound source was *P* = cos(2*πft*), and it was set to have a sinusoidal frequency. In the equation, *f* and *t* are the frequency and study time, respectively. The edge of the PVDF membrane was set as a fixed constraint; 34267 domain elements and 1803 boundary elements were formed. The study was interpreted as a time‐dependent analysis. The analysis time was set from 0 to 2000 µs with 2 µs intervals because it should be shorter than the period of applied ultrasound. The frequency of the incident ultrasound was 20 kHz, which was the same as the ultrasound generator used for the measurement. For the parametric sweep, the gap between the PVDF membrane and the Al electrode was adjusted from 0.5 to 18 mm with a 0.5 mm increment.

### Characterization

The morphology of the PVDF membrane was observed by high‐resolution field‐emission scanning electron microscopy (HR‐FESEM; SU8010, Hitachi). The crystal structure of the PVDF membrane was examined by XRD (Rigaku D/MAX 2500). The ultrasound source was generated using a commercial ultrasound generator with a power of 750 W at 20 kHz (VCX‐750, Sonics and Materials). The ultrasound power was controlled from 69 to 79 dB of the indicated intensity. The ultrasound intensity was measured using a commercial device (CRY 2301 Noise Sensor). The output voltage was measured using a high‐resolution oscilloscope (MSO46, Tektronix).

## Conflict of Interest

The authors declare no conflict of interest.

## Author Contributions

G.‐J.C., S.‐H.S., and I.‐K.P.: Conceptualization; G.‐J.C. and S.‐H.S.: Methodology; G.‐J.C. and S.‐H.S.: Formal analysis; G.‐J.C. and S.‐H.S.: Investigation; G.‐J.C., S.‐H.S., and I.‐K.P.: Writing–original draft; G.‐J.C., S.‐H.S., and I.‐K.P: Writing–review and editing.; I.‐K.P.: Supervision; I.‐K.P.: Project administration.

## Supporting information

Supporting Information

Supplemental Video 1

Supplemental Video 2

Supplemental Video 3

Supplemental Video 4

Supplemental Video 5

## Data Availability

The data that support the findings of this study are available from the corresponding author upon reasonable request.
