# Supplementary material for: Electrostatic Induction Nanogenerator Boosted by One‐Dimensional Metastructure: Application to Energy and Information Transmitting Smart Tag System
Source: Adv Sci (Weinh). 2023 Jan 22;10(11):2205141. doi: 10.1002/advs.202205141 (PMC10104663; doi:10.1002/advs.202205141)
Supplement: Supplementary file 1 — Supporting Information [file ADVS-10-2205141-s003.pdf]

ADVANCED SCIENCE
Open Access

Supporting Information

for *Adv. Sci.*, DOI 10.1002/advs.202205141

Electrostatic Induction Nanogenerator Boosted by One-Dimensional Metastructure: Application to Energy and Information Transmitting Smart Tag System

*Geon-Ju Choi, Sang-Hyun Sohn and Il-Kyu Park**

Electrostatic Induction Nanogenerator Boosted by One-Dimensional Metastructure: Application to Energy and Information Transmitting Smart Tag System

Geon-Ju Choi, Sang-Hyun Sohn, and Il-Kyu Park*

Department of Materials Science and Engineering, Seoul National University of Science and Technology, Seoul 01811, Republic of Korea

*Corresponding author. Tel.: +82-02-970-6349, fax: +82-02-973-6657

e-mail address: pik@seoultech.ac.kr (I. K. Park)

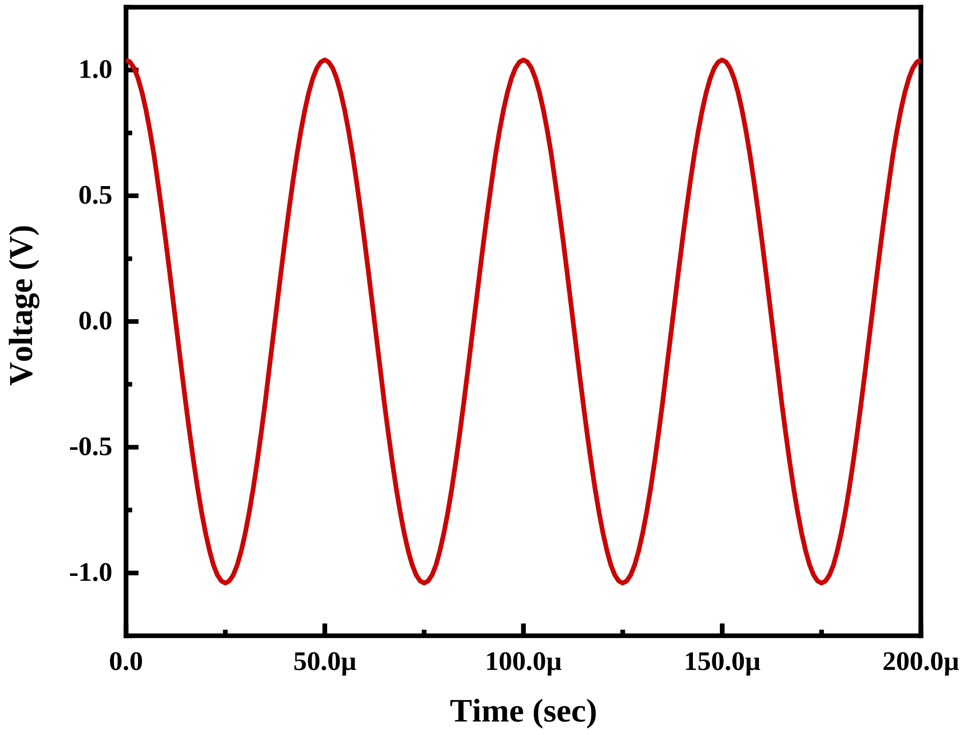

Figure S1. Voltage derived by theoretical calculations.

Figure S1. G. J. Choi et al.
