# Supplementary figures and images for: Electrostatic Induction Nanogenerator Boosted by One‐Dimensional Metastructure: Application to Energy and Information Transmitting Smart Tag System
Source: Adv Sci (Weinh). 2023 Jan 22;10(11):2205141. doi: 10.1002/advs.202205141 (PMC10104663; doi:10.1002/advs.202205141)

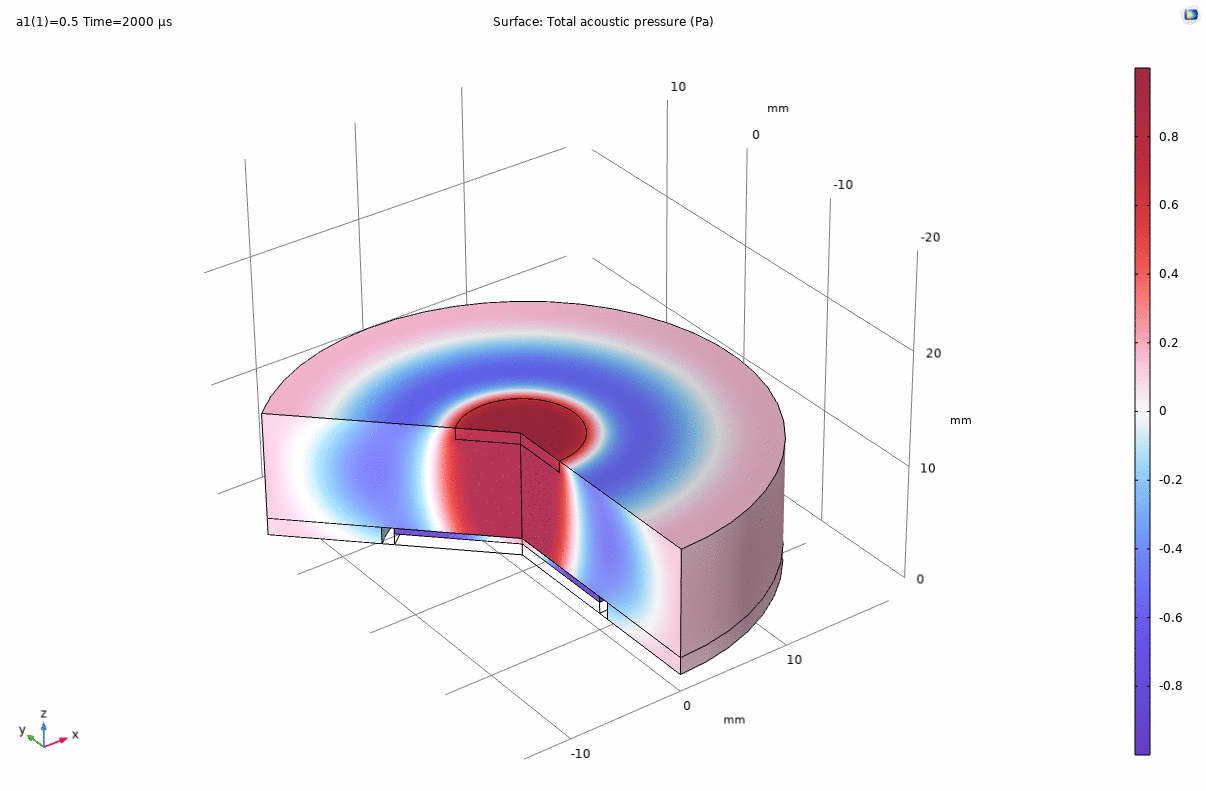

Supplement: Supplementary file 5 — Supplemental Video 4 [file ADVS-10-2205141-s001.gif]
